# Recommendations for Creating Codes of Conduct for Processing Personal Data in Biobanking Based on the GDPR art.40

**DOI:** 10.3389/fgene.2021.711614

**Published:** 2021-11-12

**Authors:** Dorota Krekora-Zając, Błażej Marciniak, Jakub Pawlikowski

**Affiliations:** ^1^ Department of Comparative Civil Law, Faculty of Law and Administration, University of Warsaw, Warsaw, Poland; ^2^ BBMRI.pl Consortium, Wroclaw, Poland; ^3^ Biobank Laboratory, Department of Molecular Biophysics, Faculty of Biology and Environmental Protection, University of Lodz, Łódź, Poland; ^4^ Department of Humanities and Social Medicine, Medical University of Lublin, Lublin, Poland

**Keywords:** code of conduct, biobanking, genetic data, recommendations, GDPR Poland

## Abstract

Personal data protection has become a fundamental normative challenge for biobankers and scientists researching human biological samples and associated data. The General Data Protection Regulation (GDPR) harmonises the law on protecting personal data throughout Europe and allows developing codes of conduct for processing personal data based on GDPR art. 40. Codes of conduct are a soft law measure to create protective standards for data processing adapted to the specific area, among others, to biobanking of human biological material. Challenges in this area were noticed by the European Data Protection Supervisor on data protection and Biobanking and BioMolecular Resources Research Infrastructure–European Research Infrastructure Consortium (BBMRI.ERIC). They concern mainly the specification of the definitions of the GDPR and the determination of the appropriate legal basis for data processing, particularly for transferring data to other European countries. Recommendations indicated in the article, which are based on the GDPR, guidelines published by the authority and expert bodies, and our experiences regarding the creation of the Polish code of conduct, should help develop how a code of conduct for processing personal data in biobanks should be developed.

## Introduction

The last few decades have seen a dynamic development of biobanks collecting human biological material and data that broaden knowledge about genetic, behavioural, and environmental determinants of many diseases, support the development of new biomarkers and drugs and improve medical care toward more personalised medicine (De Souza and Greenspan, 2013; Paskal et al., 2018; Malsagova et al., 2020). Biobanks are defined as collections of human biological material and data (McNally and Cambon-Thomsen, 2005; OECD, 2009; Taipei, 2016), and thus data collecting, processing and sharing constitute a vital part of human biological material (HBM) biobanking for scientific research purposes (Molnár-Gábor and Korbel, 2020). The processed data can sometimes make it possible to identify a natural person who submitted their material to biobanks, and so data protection in this respect poses a particular challenge (Boonen et al., 2019). That is why respecting privacy, confidentiality and data protection is among the most significant ethical and legal challenges for this activity (Towned et al., 2009; Bledsoe, 2017). The risk of privacy breach is among the most common and significant concerns reported by research participants and mentioned in public opinion surveys (Kaufman et al., 2009; Gaskell et al., 2013; Domaradzki and Pawlikowski, 2019). The strongly reverberated concerns are that the government, insurance companies, and employers could have access to such information, which might result in discrimination of the donors and their families (Porteri et al., 2014; Shabani et al., 2014). Data protection is of primary significance for building social trust, which is pivotal for the development of biobanks and their social perception (Levitt and Weldon, 2005; Toccaceli et al., 2009; Critchley et al., 2012; Domaradzki and Pawlikowski, 2019; Neethu, 2019).

The entering into force of the Regulation (EU) 2016/679 of the European Parliament and of the Council of 27 April 2016 on the protection of natural persons with regard to the processing of personal data and the free movement of such data, and repealing Directive 95/46/EC (General Data Protection Regulation, GDPR) contributed to the development of an international discussion on complete harmonisation of the personal data law protection principles in reference to various market branches. The GDPR rules shall apply to any personal data processing, regardless of its purpose, scope and processing method. The need to standardise the rules regarding natural persons’ protection was recognised much earlier, when the value of data, including personal data, increased as a subject of trade (Kuner, 2020). The acquisition of GDPR in Europe resulted in the simultaneous adoption of its rules in non-European countries (Takayuki, 2020), which facilitates data transfer between the member states and third countries (Kuner, 2020). The harmonisation is critical in scientific research, including but not limited to research on human biological samples and their related data.

It shall be emphasised that the reference harmonisation on the European Union (EU) level progresses in stages and gradually, and the GDPR was not the first community instrument in this respect (Bárd, 2009). The Directive on personal data protection of 1995 (Directive, 1995) was supposed to attain a similar objective. The legal nature and implementation method are the key differences between the Directive and the GDPR. The Directive is a legal act that sets the objectives to be achieved by the EU countries but attaining the objectives through the Directive’s implementation into the national laws depends on each country’s decision. In practice, it meant considerable differences between personal data protection systems in each country. The GDPR entered into force in all European Union states with no need to implement it. It turned out that complete standardisation of the data processing rules has not been achieved because of the generality of the GDPR provisions. Due to the fact, that the GDPR was intended as a law of general applicability that would offer protection to personal data when processed in all sectors of the EU economy there is need to further refine its provisions in the field of conducting important biomedical and genetic research (Peloquin et al., 2020). That seems why the GDPR developers predicted the possibility of further supplementing the regulation and consequently the development of bottom-up and sector-based regulations of the codes of conduct based on GDPR art. 40.

The paper aims to formulate and discuss recommendations concerning the development of a code of conduct based on GDPR art. 40, on the example of the Polish code of personal data processing by biobanks in Poland (hereinafter called the Polish code). In May 2021, the draft code (the Polish Code of Conduct) was adopted by the General Assembly of Biobanking and BioMolecular Resources Research Infrastructure in Poland (BBMRI.pl) and submitted to the President of the Polish Personal Data Protection Office.

The study should provide inspiration and assistance to code developers in other countries, including those where biobanking is already subjected to regulations of law and those where no special regulations apply.

### GDPR and Data Processing for Scientific Research Purposes in Biobanks

The GDPR concerning data protection for scientific research purposes is quite general and accounts for many inclusions for the member states. In practice, in some countries, including Poland, after the GDPR came into force, the previously applicable national regulations on data processing were discharged, which resulted in legal uncertainty as to the rules of data processing for scientific purposes. The problem turned out to be vital for biobanks, i.e., entities that collect, process and make available large databases of personal data for scientific research purposes.

Indeed, the GDPR was perceived as an up-and-coming solution. The standardisation of data exchanged fitted the open science concept and enabled the development of international research using personal data (Kaye, 2015). One of the GDPR’s objective was to promote free and safe data flow across borders. The date of the GDPR’s coming into force triggered a discussion basically in all European countries on the need to adapt national regulations to the GDPR, and the fines related to non-application of the GDPR standards resulted in the perception of personal data processing for scientific purposes as business burdened with legal and financial risks. The problem became particularly evident in biobanks which are mainly the bodies of medical universities and hospitals. Many doubts emerged as to the GDPR interpretation (Befring, 2021) and the possibility of its adaptation to the specificity of personal data processing by biobanks. The EU member states’ law referring to biobanks has been harmonised for years, e.g., developing common research infrastructures and templates of Material Transfer Agreements and Data Transfer Agreements (Chadwick and Strange, 2015).

The problems presented above can be solved by adopting the codes of conduct on the national and European level. The GDPR created the previously unknown harmonisation mechanisms such as codes of conduct. The codes became a tool to balance privacy and research interests (Hansson, 2021). They enabled the use of soft law measure for technical and organisational measures within data security and rules of data access (Shabani et al., 2021). Most importantly, it enabled the creation of sector regulation by data processing entities.

One should remember that the GDPR was developed as a protective mechanism for consumers whose data are processed for commercial purposes, and so not all the above-mentioned regulations are easily applied. The codes of conduct offered the opportunity to implement the GDPR principles for processing the data for scientific purposes in the biobanking area. The issue was also recognised in the Preliminary Opinion of the European Data Protection Supervisor (EDPS) on data protection and scientific research of 6 January 2020 (Preliminary Opinion 2020). The European Inspector for Personal Data Protection indicates that codes of conduct on data processing for scientific research purposes should be adopted in this respect. A similar approach was presented in the comments to Digital health data and services–the European health data space developed by Biobanking and BioMolecular Resources Research Infrastructure–European Research Infrastructure Consortium (BBMRI. ERIC) (BBMRI-ERIC, 2021).

It is highlighted that member states affect the GDPR’s flexibility in reference to data processing for scientific purposes (Slokenberga, 2021). On the other hand, an analysis of each member state’s legislation reveals the discrepancies in such fundamental issues as the legal basis for data processing by biobanks or the concept of public interest (Tzortzatou et al., 2021).

The codes of conduct can be developed for different purposes, depending on their application range. Generally, according to GDPR art. 40, the codes can be divided into two categories. The first category applies to the European codes based on GDPR art. 40 section 7, namely those that regulate personal data processing in several member states. Such a code, in accordance with GDPR art. 40 section 9, after the issuance of an executive act by the European Commission, becomes a generally applicable EU law. The codes are developed to harmonise the rules of personal data processing between the member states and, consequently, facilitate data transfer between EU countries. The other group includes national codes, i.e., those which regulate personal data processing on a sector level in one member state. The BBMRI. ERIC Code of Conduct for Health Research (BBMRI-ERIC, 2019) is an example of a European project concerning data processing for scientific research purposes. The Polish code of conduct can be quoted as an example of a national initiative.

The GDPR art. 89 is the critical initiative for research on biological samples, where an exception is made concerning easing the GDPR requirements on data processing for scientific purposes. According to the regulation, exceptions can be stipulated in the national law concerning data processing for scientific research purposes by limiting the right of access (GDPR art. 15), right to rectification (GDPR art. 16), right to restriction of processing (GDPR art. 18), and right to object (GDPR art. 21). Such a reference offers the opportunity for the emergence of differences between the member states in the data processing principles. It should be highlighted that the exceptions stipulated in GDPR art. 89, section 2 are acceptable only based on the national law and not the established codes of conduct. However, the code provisions fulfil an essential role because the code describe exceptions acceptable by the national law and situations in which the laws are likely to prevent or hamper the implementation for specific scientific purposes. An example of such exception the regulation of the Polish code of conduct on the right to rectification of the data included in the medical documentation can be given as an example. Essentially, such laws in Poland are limited by the provision of the Act on the Patients’ Rights and the Commissioner for patient’s rights (patient’s rights act) and the Medical Profession and Dental Profession Acts (medical profession act), and the text of the Polish code of conduct refers to those acts and describe the consequences of such regulations for biobanking.

The codes can fulfil a fairly important role for GDPR harmonisation with the national law concerning the operation of biobanks or carrying out scientific research using human biological samples. It would be an unfavourable situation to maintain different governing laws and principles applying to scientific research. It matters particularly when the domestic law is more stringent than the GDPR for personal data processing in research on human biological samples or when domestic regulations are dispersed and non-standardised (Hoppe, 2021).

### Recommendations

There are no comprehensive studies on the development of codes on conducts, including but not limited to national ones. That is why the authors would like to present their recommendations developed based on GDPR, Guidelines 1/2019 on Codes of Conduct and Monitoring Bodies under Regulation 2016/679 Version 2.0, issued by the European Data Protection Board (EDPB) (probably including the ISO standards), and our experiences from the code development). The following recommendations should be made:

1) Determination of the code application purpose and scope.
2) Determination of the minimum technical regulations for data processing safety.
3) Broad social consultations including different stakeholders’ groups.
4) Clear layout and understandable language.
5) Taking into consideration different guidelines on scientific research bioethics and ethics.

## Discussion

The development of codes of conduct complying with the GDPR art. 40 is a challenge on the European and national level. The very process of developing the codes raises many controversies. This part of the paper presents the opinions most common in the discussion on the development of codes.

### Identification of the Code’s Purpose and Application Scope

Undoubtedly the codes of conduct help particular sectors or institutions to better protect personal data according to the GDPR. The objectives resulting from the GDPR are quite general, and that is why they should be specified by the code developers, adapting the code to the specificity of the national market and the national laws’ environment. When developing the code, one should consider the specificity of the existing processing entities. The great majority of biobanks in Poland are public entities operating at medical universities and hospitals, so the code is addressed to them (Sak et al., 2012).

The sectoral nature of the code for the specific administrators’ category also results from the GDPR Recital 98. The code development aims to facilitate the GDPR application, including the adaptation of the data controllers’ and entities’ obligations to the risks of violating the natural persons’ rights or freedoms due to data processing. The details of the obligations resulting from GDPR, according to GDPR art. 40, should be provided mainly for the criteria enumerated in GDPR art. 40, section. 1, i.e., reliable and transparent processing (GDPR art. 5, section 1, letter a); legitimate interests pursued by the controllers in specific contexts (GDPR art. 6 section 1 letter f); personal data collection (GDPR art. 4 item 5); informing public opinion and the persons that the data apply to (GDPR art. 12–14); executing their rights by the persons that the personal data apply to (GDPR art. 15–23); children’s information and protection and the methods of acquiring the consent of a person with the parental authority or providing childcare (GDPR art. 8); measures and procedures mentioned in GDPR art. 24, 25 and 32; reporting any personal data breach to the supervisory body (GDPR art. 33 and 34); data transfer to third countries and international organisations (GDPR art. 44–49); dispute settlement proceedings (GDPR art. 77 and 79). The enumerated examples are only illustrative, and they shall not limit the code developers in providing broader recommendations. For instance, the Polish code includes regulations concerning dead persons’ data (it results from analyses of the Polish market (Pawlikowski et al., 2011); or particular recommendations if a biobank is closed down. The explanation lies in the fact that harmonisation of data processing principles, e.g., health or genetic data, should be among the key objectives of the codes (Phillips, 2018). In this respect, an important issue from the point of view of biobanking and conducting research on human biological material is to decide whether biological material should be treated as genetic data. In the absence of clear provisions in the Regulation concerning biological samples, one way to achieve clarity is to code of conduct. It is also necessary to emphasize that there is no unequivocal interpretation in this respect. Some point out that is since the ultimate intention of the Regulation is to protect personal data, a broad interpretation should be applied, which could allow for the inclusion of all sources, including biological samples that contain genetic data (Shabani and Borry, 2018). Others argue that due to the concept of data used by the GDPR, it is impossible to identify biological material with data (Hallinan and De Hert, 2016). Code development can be of pivotal importance for such countries as Ireland, where the previous health research laws were more liberal than the GDPR (Kirwan et al., 2021).

When determining the code’s objective, it shall be indicated whom the code should apply to, i.e., whether it should apply both to public and private entities. Public biobanks differ from private ones (Morente et al., 2017). The trend is evidenced in the scope of data processing and protection as well as their use (Quinn, 2021). The issue seems particularly relevant for national codes. Private biobanks tend to be parts of international pharmaceutical corporations, which means that they process the collected data in different member states. Their covering by the scopes of different national codes may cause constraints for harmonising data processing principles in the EU.

Moreover, social research reveals that the level of trust in private and public biobanks, both domestic and international, varies. The research participants accept data processing in their country and in public entities more than in foreign and private entities (Hung- En and Hau Tai, 2009; Masui, 2009). Although the European codes should apply to the broadest possible group of stakeholders, due to their objective of harmonising processing the data for scientific research purposes in the community, the national codes can be limited in this respect.

### Determination of the Minimum Technical Regulations for Data Processing Safety

The GDPR art. 32 is devoted to personal data processing safety aspects. The general guidelines included in the article apply to information safety management in data processing. Biobankers underestimates the importance of data security (Rychnovská, 2021) and consequently there are only a few dedicated recommendations in this area (BBMRI-ERIC, 2016; GA4GH, 2016). Therefore, the code developers should specify the general GDPR, including other international standards (e.g., ISO 27001 on information safety management system, ISO 27002 including guidelines on safety improving technical measures, ISO 27701 containing guidelines on personal data protection, and–to a minor extent–ISO 20387 that provides general requirements for biobanking) (ISO 20387; ISO 2013a; ISO 2013b) and national regulations. No general collection of rules or guidelines exists, describing the mechanisms to be implemented and how to manage them. It results from the differences between biobanks–the organisation context, legal environment, business environment and the data processing scope. That is why before adequate protection mechanisms and technical security measures are selected, a risk analysis shall be carried out. The analysis shall take into account the risks related to the processed data leak and the resultant consequences for the person whose data leaked. In addition to the biobank staff, the representatives of the unit within whose structure the biobank operates, e.g., a university or hospital, should be involved in the risk estimation. The point is to provide the persons responsible for data processing in the organisations where the biobank operates, e.g., information and communication technologies (ICT) and Personal Data Protection services, with the knowledge and potential to influence the scope and method of data processing in the biobank. The biobank operation continuity has to be ensured in the area of personal data processing, data safety backup procedures and verification of their correct execution.

ISO 2018, ISO 27000 and ISO 20387 as well as Polish regulations on the Information Safety Management Systems (Regulation of the Council of Ministers of 12 April 2012 on the National Interoperability Framework, minimum requirements for public registers and electronic information exchange and minimum requirements for the ICT systems) are included in the Polish code. The above-mentioned national regulations assume that the information safety management system ensures an adequate safety level for public administration bodies if implemented based on ISO 27001. Unfortunately, not all biobanks have the resources to manage information safety this way. That is why minimum requirements were proposed that have to be fulfilled for data processing by biobanks to be considered safe. Pseudonymisation was proposed in the Code as the primary means of securing the data. Attention was also paid to the biobank operation continuity maintenance in the personal data processing area, and guidelines were provided for the data backup procedures and verification of their correct execution. It was emphasised that the decision on the backup frequency should not result from the central plans developed regardless of the data processing place but should derive from an analysis of the risk and biobank business processes.

Moreover, the Code includes the general requirements on the safety management system, Information Technology (IT) systems used for personal data processing, data management and access, guidelines on Local Area Network (LAN) security measures, and cloud solutions. It is not only a list of requirements. Selected issues were specified in the areas that raised the most interest in the social consultation stage, and good practices were provided for each area.

The authors intended to construct the code to enable the selection of data security measures (minimum or higher level) depending on the organisation’s capabilities. The availability of adequately qualified resources, data processing scope and the analysis mentioned above were the premises for leaving the final decision on the organisation’s safety measures’ implementation. An attempt to implement too many security mechanisms at a too low staff number to handle them renders a result opposite to the expected–by dispersing the resources or assigning them to the areas that do not bear the highest safety risk.

### Broad Social Consultations Including Different Stakeholders’ Groups

According to GDPR art. 40, the associations and other entities representing specific data controller categories or data processing entities can develop codes of conduct. Organisations that associate biobanks under national and international structures naturally become the entities authorised to develop a code for biobanking (Hansson, 2021; Guidelines 1/2019). Different development rules can be adopted depending on the code. The process always consists of many stages, and social consultation should constitute its fundamental element (Guidelines 1/2019).

The BBMRI.ERIC draft code is, for instance, developed by the group responsible for its writing and then subjected to internal consultations under a Forum consisting of representatives of biobanks, organisations that associate private and public data processing entities and other stakeholders, and finally submitted for external consultation. The rules of the code development in this respect are available to the public and were the subject of many presentations in international conferences and webinars.

Guaranteeing the participation in the code development to the broadest possible group of stakeholders seems the critical issue in this respect. The consultations should involve not only the data processing entities but patients’ organisation. The code must be consulted with public authority bodies, ombudspersons and Non-Governmental Organizations (NGOs). With regard to the industry specificity, medical universities and private pharmaceutical companies should also partake in the consultations. The consultation forms should include submitting the code version for opinion, organising workshops, conferences etc. To that end, collaboration with the body that approves the code is vital. The consultations not only affect the content of the provisions but also enable broader code promotion. This applies in particular to information actions performed by the office on the codes under development. Such a collaboration facilitates control and harmonisation of actions between different entities developing the codes for related industries, e.g., health care and biobanks. They pose the most significant challenge for the code developers. Indeed, the entities participating in the consultations might have conflicting interests. This issue is controversial and suggests that a conflict between the freedom of scientific research and the right to privacy might occur in this respect (Bédard et al., 2016; Krekora-Zając, 2018; Hansson, 2021). Conducting broad and multiple social consultations, involving both patients and NGOs dealing with privacy protection, as well as entities wishing to gain access to data as much as possible seems to be the way to solve the conflict through constructive dialogue.

Broad and multi-stage social consultations were carried out for the Polish code. The initial draft code was developed under the Ethical, Legal and Societal Issues (ELSI) and IT group and was then subjected to internal consultations with the BBMRI. Pl consortium members and sent for external consultations. Between 2017 and 2020, the draft code was submitted for consultation to over thirty entities representing central administration bodies, universities, industry representatives and NGOs operating in the area of medical law, human rights and patient representation, e.g., to the Ministry of Health, the Ombudsman, National Centre for Tissue and Cell Banking, Ministry of Science and Higher Education, National Chamber of Laboratory Diagnosticians, National Pharmaceutical Chamber, Centre of Bioethics of the Supreme Medical Council, Commissioner for Patient’s Rights, Polish Bioethics Committee, Conference of Rectors of Academic Medical Universities, and NGOs [representing patients, monitoring the observance of human rights, patients’ foundations and commercial (pharmaceutical) entities]. After the draft code was translated into English, it was consulted with foreign experts working for ELSI at BBMRI.ERIC in Graz. Meetings were also held with the Personal Data Protection Office representative to discuss the code acceptance issues.

The code was presented many times in public during conferences and meetings of the Polish Biobanking Network, and it was available for the public at the bbmri.pl website (for comments). The main assumptions and essential standard solutions were presented during international and Polish conferences for the interested communities.

### Clear Layout and Understandable Language

The rule of transparency in data processing in biobanks is the supreme rule resulting from OECD guidelines, principles 1F, 1G and 1H for human material biobanking (OECD, 2009) and ISO (ISO 20387). It is also among the supreme rules of biobanks’ operation (Krekora-Zając, 2019).

In reference to the codes’ provisions, reliable and transparent processing rules should be implemented by demonstrating good practices/recommendations implying the need to determine transparent data processing procedures and informing the person whose data are processed about their data protection purpose, duration and method. It shall be emphasised that the very fact of the code development fosters the rule implementation since the code is meant to be publicly available. The code form and language are of pivotal importance in this respect. Only if the document is formulated in a way understandable for its addressees, i.e., for the entities that carry out scientific research using the data and for the research participants, will it be possible to demonstrate that the rule is followed. The use of language that is understandable for scientists who are not lawyers poses an enormous challenge for the developers of the code of conduct. A clear layout of the code contributes to attaining this objective.

In the Polish code of conduct, each chapter is divided into three units: principles, recommendations and explanations. Principles relate to legal provisions regarding the processing of personal data (resulting from the GDPR and national law). Recommendations indicate how biobanks should comply with the principle. Explanations describe how the principles and recommendations can be implemented in biobanking practice.

According to the GDPR, a code does not require a form typical of normative acts. That is why the text of the code shall include sample explanations enabling the practical application of the recommendations in the biobanking practice.

### Broad Consideration of Different Guidelines on Research Ethics

The code of conduct development within personal data processing shall also include other ethical, legal and social issues related to privacy protection in the context of human biological material biobanking. From the bioethics perspective, personal data protection is primarily related to respecting the rule of confidentiality and non-malfeasance. In the bioethics literature, attention is often paid to the scope of informed consent, access policies, biosharing, commercial use of samples and data, ownership issues, children involving, returning results or incidental findings (Pawlikowski et al., 2010; De Clercq et al., 2017; Klingstrom et al., 2018; Boonen et al., 2019; Mikkelsen et al., 2019; Prictor et al., 2019). The respective guidance is included in the Declaration of Taipei of the World Medical Association (Taipei, 2016), providing details for the biobanking area to the general ethical principles for medical research included in the Declaration of Helsinki (World Medical Association, 2013). We should be aware that the code of conduct is created to protect the people from whom the data come. Therefore, it is important to respect in the code of conduct the rights of donors, to predict procedures for cooperation with other authority when request is submitted to the data controller of biobank or to design benefit sharing system that relate to Data Processing.

Many detailed guidelines were also published by the BBMRI.ERIC (BBMRI-ERIC), Council for International Organisations of Medical Sciences (International Ethical Guidelines for Health-related Research Involving Humans, 2016), International Society for Biological and Environmental Repositories (ISBER) (2012 best practices for repositories collection, storage, retrieval, and distribution of biological materials for research international society for biological and environmental repositories., 2012), The European Data Protection Board and (Statement on the processing of personal data in the context of the COVID-19 outbreak, 2020) other organisations (Sugano and Regulatory and Ethics Working Group, 2014). Acts of the European and international law other than the GDPR regulating the ethical and legal aspects of scientific research are also vital (Convention of Biomedicine, 1997; International Declaration on Human Genetic Data, 2003; CM/Rec, 2016). The developed code should include selected regulations directly or indirectly related to data processing such as: obtaining consent, informing about data processing purpose, scope and rules, respecting the right to not to know; it may also cover the issues of informing about the research results or incidental findings management when it is related to data processing (e.g., that after data anonymization it will not be possible to provide feedback). The development of IT tools and the possibility of adapting the dynamic consent models based on them shall be considered. In ethnically diversified societies, the regulation of fair access to biobanking and research results can become a significant challenge. The rules enable regulation of the issues of processing data from vulnerable groups, e.g., children. The Polish code specifies the details of the requirements for obtaining the data processing consent, the right not to know, and processing children’s and dead persons’ data. The above bioethical issues are not directly related to art. 40 GDPR. However, these questions may be regulated in a code to improve the biobanking data processing governance.

## Conclusion

The development of the codes of conduct can improve the harmonisation of scientific data processing by biobanks. It will undoubtedly facilitate data transfer and guarantee to respect the rights of the persons that the data apply to. From a long-term perspective, it will contribute to higher trust in biobanks and research on human biological samples. In the data processing scope, the codes of conduct based on GDPR art. 40 provide an unprecedented possibility of the sector self-regulation, enabling a real influence on the adopted regulations to all stakeholders. That is why the BBMRI.Pl initiated works on the code in the area of data processing by biobanks in Poland, while BBMRI.ERIC focused on the European code. We hope that the recommendations given in the paper will inspire a discussion on the codes’ development in other European countries and accelerate the works on the European code.
